# Paroxysmal Atrial Fibrillation Catheter Ablation Outcome Depends on Pulmonary Veins Anatomy

**DOI:** 10.5935/abc.20180181

**Published:** 2018-12

**Authors:** Gabriel Odozynski, Alexander Romeno Janner Dal Forno, Andrei Lewandowski, Hélcio Garcia Nascimento, André d'Avila

**Affiliations:** 1 Universidade Federal de Santa Catarina (UFSC), Florianópolis, SC - Brazil; 2 Serviço de Arritmia e Marcapasso - Hospital SOS Cardio, Florianópolis, SC - Brazil

**Keywords:** Atrial Fibrillation/physiopathology, Arrhythmias, Cardiac, Catheter Ablation, Pulmonary Veins/physiopathology, Electrophysiologic Techniques, Cardiac

## Abstract

**Background:**

Pulmonary veins (PV) are often the trigger to atrial fibrillation (AF). Occasionally, left PVs converge on a common trunk (LCT) providing a simpler structure for catheter ablation.

**Objective:**

To compare the clinical characteristics and outcomes of ablation in paroxysmal atrial fibrillation (PAF) of patients with or without LCT.

**Methods:**

Case-control study of patients undergoing first-ever catheter ablation procedure for drug refractory PAF. The information was taken from patients' records by means of a digital collection instrument, and indexed to an online database (Syscardio(r)). Clinical characteristics and procedures were compared between patients with or without LCT (LCT x n-LCT), adopting a level of statistical significance of 5%. The primary endpoint associated with efficacy was lack of atrial arrhythmia over the follow-up time.

**Results:**

One hundred and seventy two patients with PAF were included in the study, 30 (17%) LCT and 142 (83%) n-LCT. The clinical characteristics, comorbidities, symptoms scale and risk scores did not differ between the groups. There was AF recurrence in 27% of PAF patients in the n-LCT group and only 10% of patients in the LCT group (OR: 3.4 p: 0.04) after a follow-up of 34 ± 17 months and 26 ± 15 months respectively.

**Conclusion:**

Patients with a LCT have a significantly lower recurrence rate when compared to patients without this structure. It is mandatory to report the results of AF catheter ablation as a PV anatomical variation function.

## Introduction

The electrical activity trigger responsible for triggering paroxysmal atrial fibrillation (PAF) is often located in the pulmonary veins (PV), so that the electrical isolation of the PVs is the therapeutic mainstem in the invasive treatment of this arrhythmia.^1-3^

In most patients, four pulmonary veins reach the left atrium. However, previous studies suggest that PV anatomical variations are related to a higher incidence of AF.^4,5^ The left common trunk (fusion of the 2 left PVs in a common trunk [LCT]) is the most common of the VPs anatomical variations, occurring in 4 to 18% of patients undergoing catheter ablation.^6^ However, it is not clear whether the presence of these anatomical variations changes the outcome and the recurrence rates in the invasive treatment of PAF. As LCT can be easily identified by computed tomography, knowing the clinical outcome of ablation in this population may be relevant in clinical decision-making when an ablative procedure is indicated. Therefore, the objective of this study was to compare the clinical characteristics and outcomes of patients undergoing PAF ablation with and without VPs common left trunk.

## Methods

### Study design and participants

**Figure 1:**
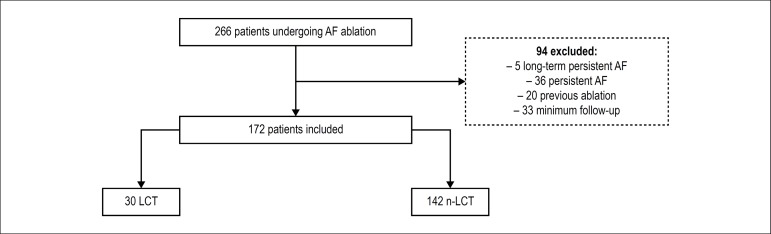
Flowchart study: patients undergoing ablation of AF categorized by presence of left trunk of the pulmonary veins.

This is a single-center, case-control study conducted between January 2011 and December 2015, with the inclusion of patients (≥ 18 years old) undergoing the first catheter ablation procedure to treat PAF that does not respond to antiarrhythmic drugs with a minimum follow-up of 12 months. The information was collected and indexed in a digital database aimed at AF ablation (SysCardio(r) software). Along with computed tomography (CT), the presence of the LCT was confirmed through a 3D atrial model constructed during the procedure with an electroanatomic mapping system by (NavX(r)). Patients with persistent or long-term persistent FA, patients with previous ablations and FA of reversible etiology, hypertrophic cardiomyopathy, rheumatic heart disease, congenital heart disease and prior catheter ablation were excluded from the sample (Figure 1).

### Procedures and ablation protocol

All procedures were performed under general anesthesia, orotracheal intubation, and invasive monitoring of blood pressure by radial or left femoral puncture, under the care of an anesthesiologist. Transseptal punctures were performed with echocardiography assistance, which was maintained throughout the procedure.

All patients underwent circumferential isolation of the PVs through a 3.5-mm tip irrigated catheter ablation without contact force measurement, using radiofrequency energy with applications of up to 35 watts and 43ºC for 30-45 seconds, and demonstration of electrical VPs entrance and exit block in relation to the left atrium at the end of the isolation. After the demonstration of entrance and exit block, patients received 18 mg of IV adenosine bolus. In cases of electrical reconnection, new mapping-guided radiofrequency applications were performed until adenosine-mediated reconnection no longer occurred. The applications in the left atrium posterior wall were performed with 20 watts for up to 15 seconds, and were interrupted in case of increased esophageal temperature > 38ºC. Applications to the left atrium posterior wall were monitored through an esophageal thermometer with multiple coated sensors (Circa(r)), and were stopped whenever there was a change in esophageal temperature above 38ºC. During all procedures performed with an electroanatomical mapping system based on thoracic impedance (EnSite Navx - Abbott(r)), IV heparin *bolus* of 100mg/kg was performed, followed by continuous infusion to keep activated coagulation time between 350 and 450s.

### Definitions of anatomical variants of the pulmonary veins

**Figure 2:**
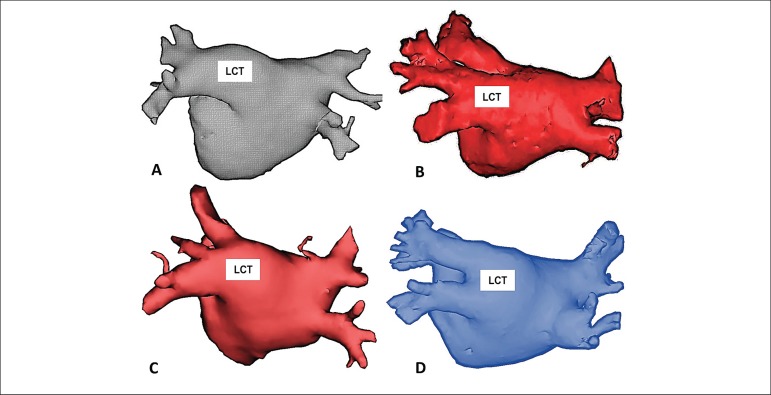
Examples of patients with Common Trunk of the Left Pulmonary Veins (LCT) obtained from Computed Tomography performed before the catheter ablation procedure. In all cases, the left pulmonary veins coalesce before insertion into the left atrium and the minimum distance between the common ostium and the beginning of the bifurcation between the lower and upper branches of the common trunk is 10 mm. All the examples are in the posterior-anterior projection highlighting the posterior wall of the left atrium.

The vein anatomy was defined as normal when two right pulmonary veins and two distinct left pulmonary veins were viewed, and the presence of the left common trunk was defined when the two left pulmonary veins coalesced on a path > 10 mm from before insertion into the left atrium in a common ostium (Figure 2).

### Clinical follow-up

After the procedure, patients remained on antiarrhythmic drugs (propafenone, sotalol or amiodarone depending on the preference of the attending physician) for 1 month, and anticoagulant for at least 3 months regardless of CHA_2_DS_2_-VASc. There was a clinical follow-up of 1, 3, 6 and 12 months after the procedure with ECG and at least two continuous 5-day electrocardiographic *(Holter)* monitoring throughout the whole clinical follow-up. At the 10th week after ablation, patients were encouraged to undergo a 5-day Holter. Any atrial arrhythmia greater than 30 seconds duration documented after 1 month of *blanking period* indicated arrhythmia recurrence.^2^ Symptoms severity before ablation and during any recurrences was characterized by the *Canadian Cardiovascular Society Severity of Atrial Fibrillation* (CCS-SAF) score, and the score of atrial fibrillation related symptoms of the *European Heart Rhythm Association (*EHRA).^7^

### Statistical analysis

Patient characteristics and procedures, recurrence rates after a single procedure, and complication rates were compared according to the groups: LCT (case) or non-LCT (control). The sample size was determined by a 1: 4 ratio for cases and controls with study power of 80%.

Continuous variables were described as mean and standard deviation and compared using unpaired Student's t-test (two-tailed), respecting the criteria of normality by Shapiro-Wilk test. Categorical variables were described by absolute number and percentages in relation to the total sample, and were compared using the χ² test or Fisher's exact test. The level of statistical significance adopted was 5%. Kaplan-Meier curve was used to evidence the relapse-free rates over the follow-up time, and Log-Rank test was used to evaluate the difference between the groups (LCT x non-LCT). Statistical analysis was performed using IBM SPSS Statistics Editor software, version 22.0.

## Results

One hundred and seventy-two patients were enrolled between 2011 and 2015 in a single center in Brazil. Thirty (17%) had LCT. There was no difference in follow-up time between cases and controls, with all patients completing a minimum follow-up of 12 months.

Table 1 summarizes the clinical characteristics of patients with LCT and non-LCT undergoing PAF ablation during the study. Variables such as age (58 ± 10 vs 62 ± 11 years), gender (71% vs 69% men), BMI (28 ± 4 vs 27 ± 3.5 kg/m²), LVEF (65 ± 8% vs 66 ± 9%), diameter of the left atrium (38 ± 5 mm vs. 39 ± 6 mm) presented no differences between the non-LCT and LCT groups, respectively. The prevalence of other comorbidities including hypertension, diabetes mellitus, coronary artery disease and risk score (CHA_2_D_2_-VASc) for stroke were similar among the samples. There was no significant difference in the severity of symptoms associated with AF (CCS-SAF and EHRA scores) between cases and controls. Four percent of the patients had a previous history of stroke.

**Table 1:** Clinical characteristics of patients undergoing AF ablation, categorization by presence of common trunk of the pulmonary veins

Variables	n-LCT (n = 142)	LCT (n = 30)	p-value
Age (years)	58.1 ± 10	62.5 ± 11	0.11
Gender (Male)	101 (71)	20 (69.2)	0.64
BMI	27.6 ± 4.5	26.6 ± 3.5	0.37
LV ejection fraction - %	64.4 ± 8.7 (33 - 86)	66.2 ± 8.5 (46 - 77)	0.33
Diameter of the LA - mm	38 ± 5.2 (27 - 53)	38.7 ± 6.3 (31 - 50)	0.76
**Comorbidities**			
SAH	82 (58.3)	18 (61.9)	0.75
DM2	16 (11)	4 (13.3)	0.54
CAD	30 (21)	4 (13.3)	0.63
Prior Stroke/TIA	6 (4.2)	1 (3.3)	0.86
CCF	10 (7)	3 (10)	0.41
CHA2DS2-VASc	1.49 ± 1.2	1.0 ± 1.9	0.49
**AF - Symptoms**			
CCS SAF score	2.07 ± 0.8	1.9 ± 0.8	0.46
EHRA score	2.04 ± 0.3	2.1 ± 0.5	0.33
**Medications**			
Statins	41 (28)	12 (40)	0.12
ACE or ARA Inhibitor	45 (31)	13 (42)	0.21
Antiarrhythmic medication	121 (85)	22 (73)	0.44

Values with ± indicate mean and standard deviation; BMI: body mass index; LV: left ventricle; LA: left atrium; SAH: systemic arterial hypertension; CAD: coronary artery disease; Stroke / TIA: stroke / transient ischemic attack; CCS SAF: Canadian Cardiovascular Society Severity of Atrial Fibrillation scale; EHRA: European Heart Rhythm Association; ACE: angiotensin converting enzyme; ARA: Angiotensin receptor antagonist 2. Student's t test and χ^2^ for independent samples.* p-value indicates a statistically significant difference at the level of 5%.

### Procedure efficacy and safety

**Figure 3:**
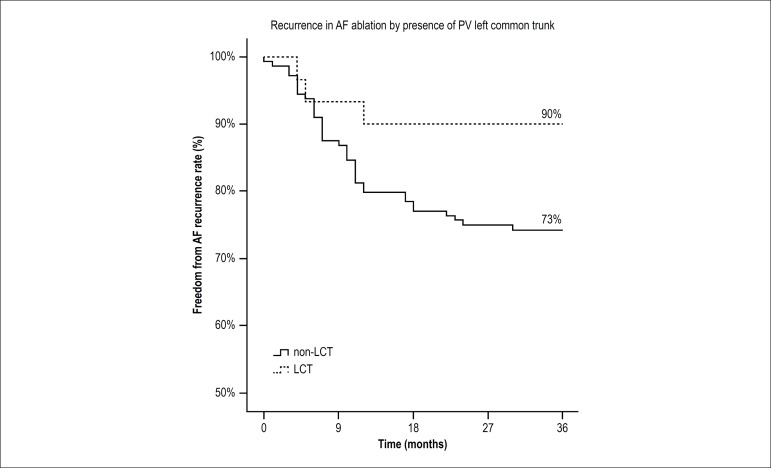
Kaplan-Meier curves for AF relapse after catheter ablation categorized by the presence of the left common trunk of the pulmonary veins; Log-rank test for comparison of recurrence curves between groups (LCT x n-LCT). p-value = 0.04

Table 2 shows a relapse rate for AF of 27% and 10% in the non-LCT and LCT groups (OR: 3.4; p: 0.04), after a follow-up time of 34 ± 17 and 26 ± 15 months respectively for cases and controls. Kaplan-Meier curve (Figure 3) highlights the lower proportion of relapse in the LCT group during the study.

**Table 2:** Efficacy of procedures and complications categorized by presence of left common trunk of the pulmonary veins

Procedures	n-LCT (n = 142)	LCT (n = 30)	OR	p-value
AF relapse	39 (27)	3 (10)	3.4	0.04*
Follow-up time	34 ± 17	26 ± 15	-	0.37
**Complications**				
Pseudoaneurysm	4 (3)	0 (0)	-	0.55
Inguinal hematoma	1 (0.7)	0 (0)	-	0.86

OR: Odds ratio; NA: not applicable; Student t test and χ^2^ for independent samples.

* p-value indicates a statistically significant difference at the level of 5%.

There were no major complications (TIA / stroke / Peripheral embolism, atrial-esophageal fistula or cardiac perforation / tamponade requiring surgery) related to procedures and / or hospitalization. Among the minor complications (inguinal hematoma, retroperitoneal bleeding, pseudoaneurysms or AV fistulas, PV stenosis, pericardial effusions or phrenic nerve palsy) there were 4 pseudoaneurysms and 1 inguinal hematoma, all in the non-LCT group, treated clinically without surgical intervention (Table 2). There were no deaths or reports of esophageal fistula during the study follow-up time.

## Discussion

The durability of PVs electrical isolation is directly related to the efficacy of the percutaneous treatment of AF, so that PVs electrical reconnection seems to be the main mechanism for post-ablation AF relapse.^2,7-10^ Our study suggests that pulmonary vein common left trunk patients have a more favorable clinical outcome after catheter ablation, with a clinical relapse around 10%. These results can be obtained without comprising procedure safety.

LCT, when present, has been indicated as the predominant origin of the triggers of AF.^11^ In the past, when the need for ablation of the 4 pulmonary veins in the same procedure was still discussed, and maneuvers were used to trigger AF, some authors suggested that when AF originated in LCT, it was not necessary to perform right PV ablation.^12,13^ Over the years, this concept proved to be inadequate because some recurrences occurred from foci in the right PVs.^2,8^ For this reason, currently the information on the presence of the left common trunk helps more in the indication of the procedure rather than in the definition of the ablation strategy, which, unless otherwise indicated, will include the ablation of the LCT and the right PVs.

In the present case-control/single-centered study of patients undergoing the first ablation procedure for PAF, it is suggested that - in comparison to the standard anatomy - the presence of the LCT favors the results in the percutaneous treatment of AF, with lower recurrence rate and low complication rates in a long-term analysis. These findings highlight the importance of knowing PVs anatomy for the efficacy and safety of AF ablation.

The definition of LCT (approximately 20% of the sample) deserves discussion. In general, the diagnosis is quite simple, through detomography or cardiac resonance. In our study, we chose an unequivocal definition of common trunk, that is, when there was a minimum distance of 10 mm between the common ostium of the left PVs and the bifurcation of their left lower and upper branches. Thus, the diagnosis of LCT was intentionally simplified. This aspect has important clinical relevance. When identifying LCT, the clinical decision for catheter ablation can be simplified because the patient with this type of anatomical change has an excellent clinical result after ablation. In fact, there was no comparison between ablation and the use of antiarrhythmic drugs in this subgroup, but it is worth remembering that all patients ablated in our study were unresponsive to antiarrhythmic drugs.

This observation has an important practical effect. In clinical practice, it is not uncommon that patients with AF and anatomically normal heart by transthoracic echocardiography undergo numerous diagnostic tests. As a rule, these patients undergo multiple stress tests, 24-hour *Holters,* and even coronary angiography with the intention of diagnosing a possible trigger for AF. Infrequently, however, these patients undergo heart CT/MRI that, if properly performed, could define the pulmonary venous anatomy and assist in the clinical decision facilitating the indication of catheter ablation.

Our study is not aligned with evidence that the presence of PVs anatomical variations (number and disposition) would be associated with a higher and more advanced degree of atrial remodeling from the electrical and structural point of view and, consequently, worse post-ablation outcome.^4,14,15^ In our results, the relapse rate in the LCT group was 3 times lower than in the non-LCT group, with patients' clinical characteristics being similar and homogeneous in all analyzes.

From the technical point of view, the presence of LCT facilitates the manipulation and the contact of the ablation catheter in the left atrium. The simpler the manipulation, the better the contact with the region to be ablated. Recent studies have shown that the tissue-to-contact relationship during ablation is crucial for lesion formation and is linked to better outcomes.^16^ Therefore, efficient tissue contact presumes a greater energy delivery through radiofrequency and formation of a more stable and homogeneous scar,^17^ which would ultimately result in a longer lasting isolation of PVs. That way, other studies using cryoablation also presented a more favorable result in patients with LCT. In cryoablation studies, however, the presence of LCT was not related to a more satisfactory outcome when compared to patients without the structure.^18^

### Limitations

The objective of this study was to describe the clinical characteristics and efficacy and safety outcomes in patients undergoing catheter ablation in the treatment of AF. This is a case-control study and, certainly, there are limitations. First, the sample size is small and may not be sufficient to detect differences between the two groups, particularly because of the low prevalence of LCT. Second, the presence of observation bias is recognized, since PVs anatomy is revealed during the procedure. Third, no detailed tool was used to assess symptoms in the presence of AF, but rather CCS-SAF and the EHRA score, which are generic scales for symptomatic evaluation. Despite this, the study provides an interesting perspective on the invasive treatment of AF in patients with LCT.

## Conclusion

In our sample, patients with LCT who underwent the first catheter ablation procedure to treat PAF presented a lower rate of relapse compared to patients without this anatomical alteration. The research on LCT should be incorporated to the investigation of PAF patients because ablation is more effective in this group of patients.
